# Application of Machine Learning to Ranking Predictors of Anti-VEGF Response

**DOI:** 10.3390/life12111926

**Published:** 2022-11-18

**Authors:** Janan Arslan, Kurt K. Benke

**Affiliations:** 1 Sorbonne Université, Institut du Cerveau—Paris Brain Institute—ICM, CNRS, Inria, Inserm, AP-HP, Hôpital de la Pitié Salpêtrière, F-75013 Paris, France; 2 Centre for Eye Research Australia, Royal Victorian Eye & Ear Hospital, University of Melbourne, East Melbourne, VIC 3002, Australia; 3 School of Engineering, University of Melbourne, Parkville, VIC 3010, Australia

**Keywords:** age-related macular degeneration, anti-VEGF treatment, explainability, statistical modelling

## Abstract

Age-related macular degeneration (AMD) is a heterogeneous disease affecting the macula of individuals and is a cause of irreversible vision loss. Patients with neovascular AMD (nAMD) are candidates for the anti-vascular endothelial growth factor (anti-VEGF) treatment, designed to regress the growth of abnormal blood vessels in the eye. Some patients fail to maintain vision despite treatment. This study aimed to develop a prediction model based on features weighted in order of importance with respect to their impact on visual acuity (VA). Evaluations included an assessment of clinical, lifestyle, and demographic factors from patients that were treated over a period of two years. The methods included mixed-effects and relative importance modelling, and models were tested against model selection criteria, diagnostic and assumption checks, and forecasting errors. The most important predictors of an anti-VEGF response were the baseline VA of the treated eye, the time (in weeks), treatment quantity, and the treated eye. The model also ranked the impact of other variables, such as intra-retinal fluid, haemorrhage, pigment epithelium detachment, treatment drug, baseline VA of the untreated eye, and various lifestyle and demographic factors. The results identified variables that could be targeted for further investigation in support of personalised treatments based on patient data.

## 1. Introduction

Research in age-related macular degeneration (AMD) can be traced back as far as 1855, according to published accounts [1,2]. For example, Donders described one of the earliest cases of AMD using microscopy and post-mortem data [2]. He noticed obliquely orientated rods that were accommodating small drusen and discovered that the rods and cones were missing above the drusen. These drusen were rarely absent in the eyes of aged individuals, especially those who were from 70 to 80 years of age. Despite many years of research into possible treatments, AMD continues to remain a progressive, chronic, and degenerative eye disease that is most prevalent in the aging population (i.e., 50 years or older) [3,4]. It is not only one of the leading causes of central and irreversible vision loss, but affected patients are at risk of developing legal blindness [5,6,7]. AMD manifests as a result of a sub-clinical inflammatory process [8] that is characterised by damage or loss of photoreceptors (i.e., cells which respond to light) and the retinal pigment epithelium (RPE; i.e., a support system for photoreceptor cells that deliver essential nutrients, such as oxygen and clear cellular debris) within the macular region [9,10].

Due to the rapid growth of the aging population, the prevalence of AMD is increasing at a significant rate [11] and is predicted to increase to 288 million by 2040 [6,12,13]. Visual impairment poses a considerable global health and economic burden due to increasing life expectancy and a growing cohort of older adults. Estimates of global vision costs for AMD were first released in 2010 which suggested a financial burden of nearly USD $3 trillion for 733 million people who were living with low vision and blindness in 2010 [14].

The disease can be broken into three sub-categories: early, intermediate, and late-stage AMD. The late-stage of AMD affects 9.64 million individuals worldwide at the time of publication, and the prevalence of late-stage AMD is predicted to increase to 18.57 million cases by the year 2040 [6]. Late-stage AMD is composed of two types: non-exudative (dry) AMD and exudative (wet) AMD; these are more commonly known as geographic atrophy (GA) and neovascular AMD (nAMD), respectively [15]. The development of GA is characterised by the death of the RPE and photoreceptor cells, as well as the closure of the underlying choriocapillaris [16]. nAMD is typified by choroidal neovascularisation (CNV), RPE or retinal detachment, retinal haemorrhage, and fibrous scarring [4,17,18]. Much of the severe vision loss occurs in the nAMD form. A Deloitte report revealed that a much greater number of patients across Australia suffered from severe nAMD than severe dry AMD [7,19]. Furthermore, the biology of nAMD is better understood as compared to GA, and thus, appropriate treatments are readily available for nAMD in the form of anti-vascular endothelial growth factor (anti-VEGF) injections designed to block and regress the growth of abnormal blood vessels in the eye that causes vision loss. The nAMD treatments include (anti-VEGF) treatments such as ranibizumab (Lucentis^®^), bevacizumab (Avastin^®^), and aflibercept (Eylea^®^). Anti-VEGF agents are injected intravitreally to stop neovascularisation [20]. While anti-VEGF treatments are available for nAMD, there have been several trials underway for other conditions [15,21]. While the response to these treatments is well-received, there remains a cohort of patients who do not respond to the treatment as expected; these patients continue to lose vision and worsen over time, potentially leading to blindness.

An exploration into the efficacy of anti-VEGF treatments can be undertaken through the evaluation of potential risk factors that trigger a lack of response. These include assessing previously implicated factors in AMD disease progression. Although age is considered the primary contributor to the development of AMD, other modifiable lifestyle risk factors, such as smoking and diet, have also been noted as important environmental insults in the progression of AMD [12,22]. Genetic risk factors are also known to play a large role in the aetiology of AMD [23]. Similarly, both modifiable and genetic factors have been implicated in the patient response to anti-VEGF treatments. Previous studies have suggested the following non-genetic factors as potential predictors in anti-VEGF response: age, baseline visual acuity (VA), the delay between symptom onset and treatment initiation, subfoveal choroidal thickness, CNV type, the location of fluid in the retina, and the presence of subretinal hyperreflective material (SHRM) [24,25,26]. There appears to be a need for continuing research relating to the hierarchy of importance of potential predictors while simultaneously producing a well-fitted prediction model to understand anti-VEGF effectiveness in AMD patients.

Biological and medical data are complex, and care needs to be taken to avoid spurious or inflated associations. There are several possible causes of confounding, including population structure (the existence of major subgroups in the population), cryptic relatedness (the existence of small groups of related individuals), and environmental factors (environmental differences between sub-populations or geographic locations) [27,28,29,30].

Several methods have been suggested to control these confounders, one of which includes mixed-effects modelling—where a set of random effects is fitted for each individual [30]. Mixed-effects models are well suited for the analysis of biological/medical data [31] and are flexible and powerful statistical models for controlling stratification, relatedness, and confounding factors [32,33,34].

A machine learning approach is investigated in the current study for the prediction of VA outcomes from anti-VEGF treatment subject to clinical data, lifestyle, and demographic factors. A variety of machine learning approaches have been the subject of past research, such as predictive regression models, including artificial neural networks, random forests, and mixed-effects models [35,36]. Many models have been applied to medical problems in ophthalmology but have limited explainability [37,38,39]. A challenge to machine learning is to develop models that are not black box in nature but incorporate explainability in their predictions. In this study, a machine learning approach was developed that incorporates statistical features and metrics to produce a degree of explainability.

Potential predictor variables can be ranked by weights in the order of importance using the relative importance of variables (RIV) method. Larger predictor weights are considered the most important, while those with smaller weights are considered the least important [40].

This paper has two objectives: (1) to apply machine learning to develop mathematical models to predict vision outcomes for anti-VEGF-treated AMD patients; and (2) to rank variables that are available to the ophthalmologist, in order of importance (i.e., largest to smallest weights). The best models were selected based on model selection techniques, along with diagnostic and forecasting evaluations. The aim was to develop a prediction model to include the features most responsible for treatment response and to optimise prediction accuracy.

## 2. Materials and Methods

### 2.1. Study Design

A retrospective analysis was conducted as a case study using anonymised data from patients who attended the retina clinics at the Royal Victorian Eye and Ear Hospital (RVEEH). The study was approved by the Human Research Ethics Committee of RVEEH. The study was conducted in accordance with the International Conference on Harmonisation Guidelines for Good Clinical Practice and tenets of the Declaration of Helsinki Ethics approval was provided by the Human Research Ethics Committee (HREC: Project No. 95/283H/15) by the RVEEH. Written informed consent was obtained from all participants.

### 2.2. Patient Data

The patient dataset consisted of 150 treatment-naïve eyes, with patients >50 years of age who were diagnosed with subfoveal CNV secondary to AMD and who had attended the RVEEH between 2006 and 2010. Clinical diagnoses were based on a retinal examination, fundus photography, fundus fluorescein angiography, time-domain optical coherence tomography (OCT) with Stratus OCT version 5.0.1 (Carl Zeiss Meditec, Dublin, CA, USA) or Cirrus HD-OCT version 6.0.0.599 (Carl Zeiss Meditec). VA scores were obtained using the early treatment diabetic retinopathy study (ETDRS) chart performed at 4 m. The presence of intra-retinal fluid (IRF), sub-retinal fluid (SRF), macular thickness, macular scar, atrophy, and haemorrhage were analysed using OCT. Results were collated for baseline at three, six, twelve, and twenty-four months treatment intervals.

Patients with CNV secondary to non-AMD conditions, such as angioid streaks, severe myopia, central serous retinopathy, or hereditary retinal disorders, and those who received any previous treatment for nAMD, such as an anti-VEGF, photodynamic therapy, or laser photocoagulation were excluded.

### 2.3. Data Format

The time-series data followed the treatment schedule and clinical manifestations of all 150 eyes over the course of a two-year treatment. The dataset included general demographic information, such as age, gender, and ethnicity, along with several clinical variables (Table S1, Supplementary Materials). We identified whether each variable was binary, categorical, or continuous as part of our exploratory analysis.

The data were initially presented in the “wide” format, which contained approximately 156 variables across all 150 eyes. The data were converted into a “long” format, amalgamating variables across multiple time points into a single variable. For example, rather than having five variables for the VA at baseline at three, six, twelve, and twenty-four months, a single VA variable with a time variable as a reference was used.

### 2.4. Treatment Protocol

Patients were treated with either ranibizumab or bevacizumab, with most receiving ranibizumab, where bevacizumab was used occasionally for the first injection whilst awaiting approval for the subsidised use of ranibizumab (aflibercept was not available at the time). A total of 140 patients were treated for either the left eye (LE) or the right eye (RE), and five patients had both eyes treated. All patients received 3 initial monthly injections followed by a flexible (as required) period. The decision to re-treat in the flexible period was at the discretion of the treating retinal specialist at each follow-up visit on the basis of re-treatment criteria, including the VA loss of 5 letters, increased central retinal thickness of 100 µm, or the presence of retinal fluid on OCT (intraretinal or subretinal) or ophthalmic examination findings of new or persistent haemorrhage. The extension of 2 weeks was considered for the subsequent clinic visit if the clinical situation was stable and OCT was free of intra-retinal or sub-retinal fluid. This evolved into a treat-and-extend protocol in the latter half of the time period, where if the patient showed no signs of activity, the time between the injections was extended by two weeks. Individuals with persistent signs of activity continued to receive monthly injections.

### 2.5. Statistical Analysis

All statistical analyses were run using the statistical software R version 3.2.2. [41]. The null hypothesis for the RIV analysis was that the parameter estimates for all variables were identical and had the same level of importance in their contribution to vision outcomes in anti-VEGF-treated AMD patients.

#### 2.5.1. Modelling Mixed-Effects

Mixed-effects models are used to describe relationships between response and predictor variables in data that are grouped based on one or more classifications [42]. Mixed-effects models explicitly specify the mean and covariance structure, incorporating two types of parameters: fixed and random effects [43,44]. Fixed effects refer to predictors that affect a response variable. Random effects, however, refer to effects on a response variable generated by variation within and among the levels of a predictor variable [43]. Population structure is the fixed effect in a mixed-effects model, while relatedness among individuals is incorporated as a variance-covariance structure of the random effect [45]. Mixed effects models have gained considerable popularity and are considered useful in the analysis of longitudinal data, the modelling of complex clustered data, penalised log-likelihood, etc. [31,46]. There are advantages to using mixed models in medical applications.

A medical study may be carried out at multiple locations, clinics, or hospitals, and therefore, medical data may often be clustered. The design of a medical study may be described as hierarchical and wider inferences can be made by fitting the clustering effect as a random effect. Repeated measurements are also common in medical studies, and it is not uncommon for several observations to be missing. The advantage of using a mixed-effects model is that it makes allowance for missing data and hierarchical clustering [47].

The RVEEH dataset is from a longitudinal study and consists of repeated observations by individual subjects over a time series. The research interest lies in the effects that are common and different among all individuals in the study [48]. The mixed-effects model allows the capture of among-subject variations. The use of mixed-effects modelling is that it assists in explaining variability in the patient response to anti-VEGF treatment and helps to identify other factors that may contribute to treatment response.

Linear mixed-effects models are an extension of regular linear models. Traditional linear models use only a single random term, the residual error. A linear mixed-effects model allows the specification of more than one random term [49], a useful feature, as it is more accurate to think of an effect coming from a specific normal distribution rather than that of a fixed value [50].

With *N* independent sampling units (i.e., the patients), the linear mixed-effects model for the ith person may be written as follows:

(1) $$Y_{i}=X_{i}\beta_{i}+Z_{i}u_{i}+\varepsilon_{i}$$

where $Y_{i}$ represents the response variable for the ith person, $X_{i}$ is a $n_{i}\times p$ design matrix for the p-vector of the fixed effects β, and $Z_{i}$ is a $n_{i}\times q$ design matrix associated with the q-vector of random effects $u_{i}$ that represent subject-specific regression coefficients. The error term, $\varepsilon_{i}$, is assumed to be normally distributed with a mean zero and to be independent of the random effects [51].

The use of linear mixed-effects models counters the multiple drawbacks that are normally associated with traditional random effects modelling, such as [52]:

(a) Deficiencies in statistical power with the use of repeated observations;
(b) Lack of adaptability around dealing with missing data;
(c) Disparate methods for treating continuous and categorical responses;
(d) Unproven methods for modelling heteroscedasticity and non-spherical error variance.

There are multiple measurements for each subject thus, we need to incorporate random effects into the model to account for the variation in outcomes. To account for within-subject dependencies, a subject-specific latent variable (i.e., random effects) must be included in the model. Typically, an additional random effect is included for each regression coefficient that is expected to vary among the subjects. For example, in dose–response settings, one may account for baseline heterogeneity through a random intercept and for heterogeneity in susceptibility through a random slope, with these two factors potentially correlated [53]. To account for this heterogeneity, the random effect used across all our tested models included *time* (in weeks) and *subject*. This is represented in the analysis as (*time*|*subject*). The use of the random effect *subject* accounts for the random intercept. The random effect *time* accounts for the random slope. Software for data analytics was developed for this project and also sourced for linear mixed-effects models from the work of Bates and Maechler, as maintained by Ben Bolker [54].

#### 2.5.2. Measure of Outcome

For our response variable, we preferred the use of follow-up VA measurements for both the LE and RE as the outcome/dependent variable (i.e., $Y_{i}$). All remaining variables, including the baseline VAs, were considered potential predictors. The responses were additionally divided into LE and RE.

Sometimes, change scores (i.e., post-treatment outcomes minus pre-treatment measurements) were used in place of follow-up scores as a way of accounting for chance imbalances at the baseline between treatment groups. Baseline imbalances can include factors such as age or disease severity; they can occur either due to (i) a true biological variability within the individual, or (ii) due to a measurement error, or even a combination of the two [55,56]; these imbalances are referred to as a regression to the mean [57]. While it may seem intuitive to use change scores to control for any chance imbalances at the baseline, as outcomes may occur due to regression to the mean, we opted to use follow-up scores in place of change scores instead.

#### 2.5.3. Model Selection

The information criteria, such as the *Akaike Information Criterion* (AIC) and *Schwartz* or *Bayesian Information Criterion* (BIC), were used in the model selection process. Although a plethora of information criteria are available for model comparison, they are modifications or generalisations of the AIC or BIC [58]. The AIC and BIC criteria are defined as [59]:

(2) $$AIC=2\left[\ell \left({\hat{\theta }}_{2}\right)-\ell \left({\hat{\theta }}_{1}\right)\right]-2\left(p_{2}-p_{1}\right)$$

(3) $$BIC=2\left[\ell \left({\hat{\theta }}_{2}\right)-\ell \left({\hat{\theta }}_{1}\right)\right]-\log_{n}\left(p_{2}-p_{1}\right)$$

where $2\left[\ell \left({\hat{\theta }}_{2}\right)-\ell \left({\hat{\theta }}_{1}\right)\right]$ is the likelihood ratio test statistic that is asymptotically distributed as $\chi^{2}$ with $p_{2}-p_{1}$ degrees-of-freedom. AIC and BIC theories have the same objective: to find the best model via comparison. However, each theory has a different motivation. While AIC compares models using a measure of similarity in the expected predictive performance, BIC compares the probabilities that each of the models tested is the true model [58].

The main idea behind the selection criteria is to compare models based on their maximised log-likelihood value, while penalising for the number of parameters. The model with the smallest AIC or BIC values is deemed the best [60]. Additionally, in finding the smallest AIC and BIC values, the model chosen needs to provide a good fit to the data, using $R^{2}$, also known as the *coefficient of determination,* which relates to the impact of the predictor variable *X* [61]. Values for $R^{2}$ range from $0\leq R^{2}\leq 1$. Values closer to 1 indicate a better fit.

For mixed-effects models, $R^{2}$ can be categorised into two types: marginal $R^{2}$ and conditional $R^{2}$. Marginal $R^{2}$ accounts for the variance explained by fixed factors:

(4) $$R_{M}{}^{2}=\frac{\sigma_{f}^{2}}{\sigma_{f}^{2}+\sum_{l=1}^{u}\sigma_{l}^{2}+\sigma_{e}^{2}+\sigma_{d}^{2}}$$

and conditional $R^{2}$ is concerned with the variance explained by both fixed and random factors [27]:

(5) $$R_{C}^{2}=\frac{\sigma_{f}^{2}+\sum_{l=1}^{u}\sigma_{l}^{2}}{\sigma_{f}^{2}+\sum_{l=1}^{u}\sigma_{l}^{2}+\sigma_{e}^{2}+\sigma_{d}^{2}}$$

where $\sigma_{f}^{2}$ = the variance calculated from the fixed effects component; u = the number of random factors in the model; $\sigma_{l}^{2}$ = the variance component of the lth random factor; $\left(\sigma_{e}^{2}+\sigma_{d}^{2}\right)$ = the sum of an additive dispersion component and the distribution-specific variance.

#### 2.5.4. Model Diagnostics

Once a suitable model has been identified and fitted, the key assumptions of the model can be tested. These assumptions include (i) linearity, (ii) homoscedasticity or constancy of the error variance, and (iii) normality of the errors. Discrepancies between the assumed model and data can be identified by studying the residuals (also known as the error component). The residuals represent the differences between observed and predicted values for the assumed model. Visual aids, such as residual plots, help identify whether the assumptions of the model have been satisfied. Typically, a good residual plot would be one with an even horizontal distribution of residuals or symmetry; whereas those that contain distinguishable patterns, such as being clustered to one side of the plot, usually indicate a violation of the model assumption and warrant a further review of the model (e.g., appropriate transformation of dependent or independent variables) [62]. Normal probability plots additionally allowed us to determine the fit of our model.

Using both residual plots and normal probability plots, we could identify any unusual or outlying observations based on large deviations in the observed Y values from that of the fitted line. Inferences drawn from the model can be potentially influenced by only a few cases in the data. The fitted model may reflect the unusual characteristics of those cases rather than the overall relationship between the dependent and independent variables [63].

Influence analysis consists of investigating whether observations (or a group of observations) are given disproportionate importance in the model estimation. The simple inclusion or exclusion of an influential case may lead to substantially different regression estimates [64]. *DFBETAS* is a standardised measure that indicates the level of influence observations have on single parameter estimates [65]. For mixed-effects models, this relates to the influence of a higher-level unit on the parameter estimates. *DFBETAS* is calculated as the difference in parameter estimate between the model included and the model excluding the higher-level case. This absolute difference is divided by the standard error of the parameter estimate excluding the higher-level case [66]:

(6) $$DFBETAS_{ij}=\frac{{\hat{\gamma }}_{i}-{\hat{\gamma }}_{i\left(-j\right)}}{se\left({\hat{\gamma }}_{i\left(-j\right)}\right)}$$

in which i refers to the parameter estimate and j the higher-level group, so ${\hat{\gamma }}_{i}$ represents the original parameter estimate i, and ${\hat{\gamma }}_{i\left(-j\right)}$ represents the estimate of the parameter i after the higher-level group j has been excluded from the data. We used the *influence.ME* package in R to run these analyses [66]. As a rule of thumb, the cut-off value for *DFBETAS* is given as [67]:

(7) $$\;CoV=2/\sqrt{n}$$

in which n is the number of observations under evaluation. Values exceeding this cut-off are regarded as potentially influencing the regression outcomes for that specific estimate.

As *DFBETAS* provides a value for each parameter and for each higher-level unit that is evaluated, this can result in a large number of values to review. An alternative method for identifying influence is Cook’s distance. Cook’s distance provides a summary of measures for the influence that a higher-level unit exerts on all parameter estimates simultaneously. A formula for Cook’s distance is [66]:

(8) $$C_{j}^{OF}=\frac{1}{r+1}\left(\hat{\gamma }-{\hat{\gamma }}_{\left(-j\right)}\right)\prime \hat{\sum }\left(\hat{\gamma }-{\hat{\gamma }}_{\left(-j\right)}\right)$$

where $\hat{\gamma }$ represents the vector of the original parameter estimates ${\hat{\gamma }}_{\left(-j\right)}$ the parameter estimates of the model excluding the higher-level unit j, and $\hat{\sum }$ represents the covariance matrix. As a rule of thumb, cases are regarded as potentially influential if the associated value for Cook’s distance exceeds the cut-off value of [68]:

(9)  CoV=4/n

where n refers to the number of groups in the grouping factor under evaluation.

To test for changes in statistical significance, we employed the *sigtest()* function. This is used to test for changing levels of significance after the deletion of each of the potentially influential data points identified using *DFBETAS*. For the Cook’s distance, we carried out similar functions using the *exclude.influence()* function. While there could be many potentially influential points, those that created statistically significant changes upon deletion were considered overly influential.

#### 2.5.5. Prediction Accuracy

Past data allows the identification of a pattern that can be extrapolated or extended into the future in order to prepare a prediction or forecast. Forecasting techniques rely on the assumption that the patterns which have been identified in the past will continue in the future. Good predictions cannot be expected unless this assumption is valid. Forecasting is subject to uncertainty analysis. There may be an irregular component that may be substantial and cause fluctuations in the data. Hence, we reviewed forecasting errors in an attempt to ascertain whether an irregular component was so large as to completely invalidate any forecasting technique or perhaps the forecasting technique used was not capable of accurately predicting the trend, seasonal, or cyclical components of the data, thus rendering the technique inappropriate [69].

The first metric to assess forecast quality is the mean error (*ME*), which is simply the average of past errors between the *n* observed and forecast values:

(10) $$ME=\frac{1}{n}\sum_{t=1}^{n}e_{t}$$

where we used the following notation [70]:

$e_{t}=Y_{t}-{\hat{Y}}_{t}$ is the forecast error for a particular at time t;

${\hat{Y}}_{t}$ = the forecast value generated in period t (i.e., the fitted/predicted value);

$Y_{t}$ = the observed value at time t.

The *ME* metric reveals whether the forecasting process, on average, tends to under-forecast (i.e., *ME* would be positive) or over-forecast (i.e., *ME* would be negative); it was, in fact, a metric of bias. We, therefore, needed other metrics for forecast accuracy that could capture the proximity between the prediction produced using our model and the actual observed values.

The first metric for forecast accuracy is the mean absolute deviation (*MAD*). *MAD* uses the absolute error to ensure that negative and positive errors do not cancel when averaged:

(11) $$MAD=\frac{1}{n}\sum_{t=1}^{n}\left|e_{t}\right|$$

The second metric for forecast accuracy is the root mean square error (*RMSE*)—this measure squares errors to the sum of positive and negative ones. The *RMSE* is similar to the standard deviation (except that the deviations are not around the mean value):

(12) $$RMSE=\sqrt{\frac{1}{n}\sum_{t=1}^{n}e_{t}^{2}}$$

The previous metrics are measured in the same units as the data and are not scale-independent. The normalisation of accuracy requires expression as a proportion or percentage. The metrics which accommodate for this are the mean percentage error (*MPE*) and mean absolute percentage error (*MAPE*), which measure percentage bias and percentage accuracy, respectively.

Our objective was to find a model that would have a prediction error rate of less than 10% (i.e., our prediction accuracy was not off by more than 10%):

(13) $$MPE=\frac{1}{n}\sum_{t=1}^{n}\frac{e_{t}}{Y_{t}}$$

(14) $$MAPE=\left(\frac{1}{n}\sum_{t=1}^{n}\frac{\left|e_{t}\right|}{Y_{t}}\right)\times 100$$

As these measures are percentages, no further scaling is required and interpretation is straight forward [69].

#### 2.5.6. The RIV Method

The RIV method ranks predictor variables by weights, where larger predictor weights are considered more important, while those with lower weights are considered less important [71]. The advantage of this method is that it ensures that the variables are not evaluated as if all are equally important. By appropriate variable weighting, our model can determine which factors will have the most influence on the outcome. The ranking and weighting of variables improves the model accuracy, as the weighting reflects the contribution of each parameter to the outcome. A package for AIC determination was used to identify the level of importance for each variable using the RIV method [72].

To estimate the RIV of variable $x_{j}$, the sum of all Akaike weights is required (i.e., AIC) across all the models in the set where j occurs; the sum of $w_{+}\left(j\right)$ reflects the importance of the variable. This sum is denoted as a numerical value between 0 and 1. The larger the sum $w_{+}\left(j\right)$ (i.e., closer to 1), the more important the variable is relative to other variables tested. Using $w_{+}\left(j\right)$, all the variables can be ranked in order of their importance.

The effect size is based on model-averaged estimates. It is, therefore, important to ensure a balance in the number of models which include the variable j. In other words, to ensure an accurate reflection of the importance of one variable versus another, a combination of models is required, which contain all prospective variables in equal proportion across all models, allowing each variable to be tested on an equal footing. Otherwise, if one variable were to be found more frequently across our test models, as compared to another, it may inadvertently give the more frequently occurring variable the advantage.

Typically, to calculate the Akaike weights, the following formulae are used:

(15) $$\mathrm{AIC}=-2\log\left(L\left(\hat{\theta }|data\right)\right)+2K$$

(16) $${∆}_{i}={\mathrm{AIC}}_{i}-{\mathrm{AIC}}_{min}$$

(17) $$L\left(g_{i}|data\right)=\exp\left(-\frac{{∆}_{i}}{2}\right)={\mathrm{likelihood}\;\mathrm{of}\;\mathrm{model}\;g}_{i}$$

(18) $$w_{i}=\frac{\exp\left(-\frac{{∆}_{i}}{2}\right)}{\sum_{r=1}^{R}\exp\left(-\frac{{∆}_{r}}{2}\right)}$$

Alternatively, the weights can be viewed as a proportion of evidence,

(19) $$w_{+}\left(j\right)=\sum_{i\;\mathrm{for}\;X_{j}\in g_{i}}w_{i}$$

which is the sum of the model weights for the subset of the models that contain the predictor variable $x_{j}$. The sum of the models for the subset of all the models that *did not* contain the predictor variable $x_{j}$ is:

(20) $$w_{-}\left(j\right)=\sum_{i\;\mathrm{for}\;X_{j}\notin g_{i}}w_{i}$$

Hence, the importance of predictor $x_{j}$ is associated with the contrast between $w_{+}\left(j\right)$ and $w_{-}\left(j\right)$, with $w_{+}\left(j\right)+w_{-}\left(j\right)=1$. The larger the $w_{+}\left(j\right)$ value is, the more important the predictor $x_{j}$.

#### 2.5.7. Treatment of Missing Data

Generally, mixed-effects models are more flexible in the treatment of missing data than fixed-effects models. It is reasonable to assume that a mixed model is capable of handling the imbalance caused by missing observations, provided that the data points are missing at random. When data cannot be considered to be missing at random, ad hoc approaches, such as the “last value carried forward” (i.e., where the last observed value of the response variable is substituted for every subsequent missing observation), are used [47].

For the selection of the mixed-effects model, we opted to use two methods to correct for missing data: the multiple imputation (MI) method to identify potential predictor variables and the stacked MI method to validate (or possibly further investigate) our original findings. For the RIV analysis, we simply used the MI method. Both methods aimed to restore the dataset from its incomplete state to that of completeness by substituting reasonable estimates for each missing data point.

The MI method, which was proposed by Rubin in 1978, rectifies the major disadvantage of single imputation—the under-representation of uncertainty [73,74,75]. While MI has the appeal of restoring the full dataset, we realise that there is no way to recover the actual unknown missing values. It is, therefore, important to note that imputed datasets are not to be treated as substitutes for true completed datasets but rather designed to produce valid overall inferences from the original incomplete dataset [76]. 

#### 2.5.8. The Multiple Imputation (MI) Method

Generate an m number of copies of the incomplete dataset, using an appropriate procedure to impute the missing values in each copy. As we do not know the true values, the imputed values used in each copy are different from each other. The m values are ordered in the sense that the first components of the vectors for the missing values are used to create one completed data set, the second components of the vectors are used to create the second complete data set, and so on. Each completed dataset is analysed using standard complete-data methods [77]. The repetition of m  times accounts for variability due to unknown values [78,79]. We opted to produce m = 5 imputed datasets, producing five separate (and complete) datasets, each with 150 rows of data.

(a) For each imputed copy of the dataset, standard analysis is performed, and the parameter estimates of interest are stored.
(b) Using “Rubin’s rules”, a combined estimate of the parameter is generated as the average of the m separate estimates [76].

Step 1, the imputation step, predicts or fills in the missing values multiple times using the conditional distribution of the observed data. Although several imputation methods exist, such as predictive mean matching, the Markov Chain Monte Carlo (MCMC), or chained equations, the preferred method is one that matches that missing data pattern [80].

In the process of model selection, the MI method generally yields different predictor variables across each dataset. Three strategies have been proposed which “combines” and identifies the single most suitable model across all imputed datasets [81]:

(a) Select predictors that appear in any model;
(b) Select predictors that appear in at least half of the models;
(c) Select predictors that appear in all of the models.

In this study, it was found that the second of the proposed methods was preferred, as it allowed us to find commonalities between each imputed dataset and provided the flexibility to assess the discrepancies in variables that appeared infrequently across all the datasets.

We additionally used the stacked weighted regression method to validate the model findings using the MI method. Rather than reviewing each imputed dataset separately, the five imputed datasets were “stacked” to create one large dataset of length m×n in place (m imputed datasets for n individuals). While fitting models to single-stacked data yields and valid parameter estimates, standard errors may end up being too small. To correct this issue, we scaled the log-likelihood for the stacked data using weights in our regression models, which additionally accounted for the degree of missing information in the dataset:

(21) $$w_{i}=\frac{1-f}{m}$$

where f is the fraction of missing data across all variables—the total number of missing data divided by np, with n being the number of individuals (150), and p is the number of predictor variables (19) [81,82].

**Figure 1:**
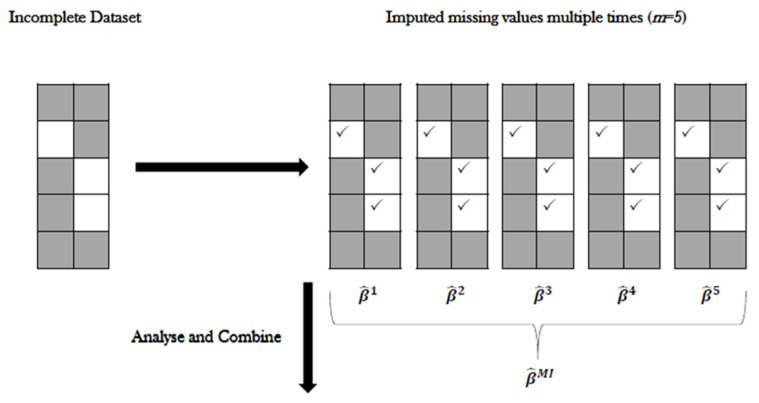
Schematic illustration of multiple imputation method (adapted from Molenberghs et al, 2015 [76]). This illustration demonstrates the imputation of an incomplete dataset. Each dataset was then analysed and the results were combined. (√) refers to imputed portions of dataset.

**Figure 2:**
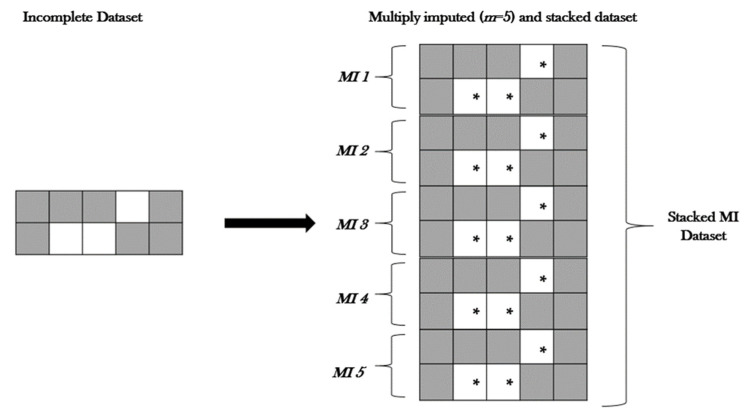
Schematic illustration of multiple imputation and stacked dataset method. This illustration demonstrates the imputation of an incomplete dataset five times. The imputed datasets are then “stacked” together to form one large dataset. Rather than carrying out multiple analyses and combing the results, this method allows the analysis of one single dataset. * refers to imputed portions of dataset.

For both our MI and stacked MI methods, we used the R package Amelia [83]. Amelia resamples the original data using a bootstrap algorithm while implementing an expectation-maximisation (EM) algorithm—an iterative method for maximum likelihood or maximum a posteriori estimates [84]. Amelia uses all observed data to estimate the missing values, then creates several complete datasets that include the original data points plus slightly different imputed points to account for uncertainty (Figure 1). For stacked MIs, the same method of imputation takes place, with the addition of including the command separate = FALSE to ensure the imputed datasets are not separated and kept as one (Figure 2).

## 3. Results

### 3.1. Summary Statistics

Our cohort of 150 eyes consisted of 85 eyes (56.7%) from females and 65 eyes (43.3%) from males (Table 1). The mean age, with standard deviation (SD) at the baseline, was 78.9 ± 7.3 years. The mean baseline VA for the LE was 53.5 ± 24.0 letters, while the RE was 48.4 ± 24.3. At the baseline, ranibizumab was injected 122 times (81.3%) and bevacizumab 28 (18.7%).

Most patients were treated for the RE, with 86 (57.3%) patients being treated in the RE, while 64 (42.6%) were treated in the LE. Ten (6.7%) patients were treated in both the LE and RE. A total of 102 (68%) patients had hypertension, and 25 (16.7%) had diabetes.

#### Missing Values

**Figure 3:**
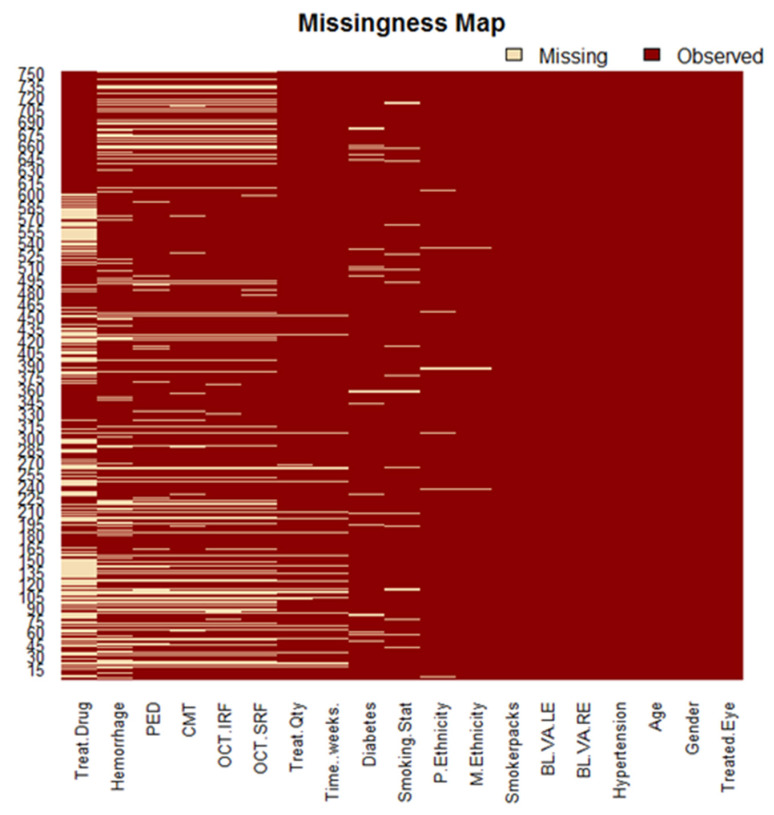
Missing map for original dataset. The map illustrates missing values across all variables tested for the treatment duration of 24 months. Those marked with dark red represent observed and available data, while the light pink represents missing data. Most of the missing information can be found in OCT derived variables. We found that treatment drug had the most missing values (35.6%), followed by haemorrhage (24.8%), and PED (20.67%).

Figure 3 displays a Heatmap which highlights missing values. Variables with no missing data included: the treated eye, age, gender, hypertension, smokerpacks, and baseline VA. Variables with a few missing data included: paternal (2%) and maternal ethnicity (1.33%), smoking status (4%), diabetes (4.67%), time (in weeks) (4.8%), and treatment quantity (5.2%). Finally, variables that contained a substantial amount of missing values included: OCT derived SRF (18.13%), IRF (18.27%), CMT (19.47%), PED (20.67%), haemorrhage (24.8%), and the treatment drug (35.6%). We assumed that greater variability in our outcomes would be found in the last set of variables and anticipated consistent results for all other variables.

### 3.2. The Mixed-Effects Model

#### 3.2.1. Identifying Predictor Variables

We tested for all possible combinations of all 19 predictor variables (i.e., 524,288 models, including null models) for each imputed dataset and stacked imputed datasets. Possible combinations were tested in the following format:

1. Inspect the ith combination of predictor variables;
2. Add the ith combination into a mixed-effects formula, which includes the random effects variables for *time* and *subject;*
3. Store the AIC;
4. Store the BIC;
5. Once all possible combinations have been tested, list the combinations that produce the smallest AIC and BIC values.

Each tested model followed the format below:

*Response = ith combination of predictor variables + random effects* (22)

Using the MI method with five separate datasets (Table S2, Supplementary Materials), we initially identified the following predictors as producing the models with the lowest AIC/BIC for both the LE and RE.

In our methods (*Treatment of Missing Data*), our process of selecting the most appropriate predictors for a model included finding variables that appeared in at least half of the imputed dataset outcomes, with the flexibility to explore other predictor variables that occurred less frequently.

We then proceeded to repeat our analysis using the single stacked imputed dataset (Table S3, Supplementary Materials) in place of five separate imputed datasets.

Following the results from both methods, we proceeded to test models that included any of the predictors included in Tables S2 and S3 in the Supplementary Materials. The final model choice was additionally based on: (1) diagnostic outcomes and (2) prediction accuracy. The following model for both the LE and RE provided the most consistent prediction outcomes, in line with model assumptions:

(23) $$Y_{i}=\beta_{0}+\sum_{j=1}^{j=10}\beta_{j}X_{j}+Z_{i}u_{i}+\varepsilon_{i}$$

$Y_{i}$ = VA at time t  (LE or RE); $X_{1}$ = LE baseline VA; $X_{2}$ = RE baseline VA; $X_{3}$ = OCT IRF; $X_{4}$ = OCT CMT; $X_{5}$ = time (in weeks); $X_{6}$ = treatment quantity; $X_{7}$ = treatment drug; $X_{8}$ = treated eye; $X_{9}$ = OCT haemorrhage; and $X_{10}$ = OCT PED.

While other potential variables such as age, hypertension, and OCT SRF were also tested, it was found that the addition of these variables to the model neither added nor subtracted from the accuracy of the model. The preference was for an efficient model, with the least variables needed to produce an accurate outcome and to guard against over-fitting with the ten selected variables forming the basis of the final model.

#### 3.2.2. Model Diagnostics

**Figure 4:**
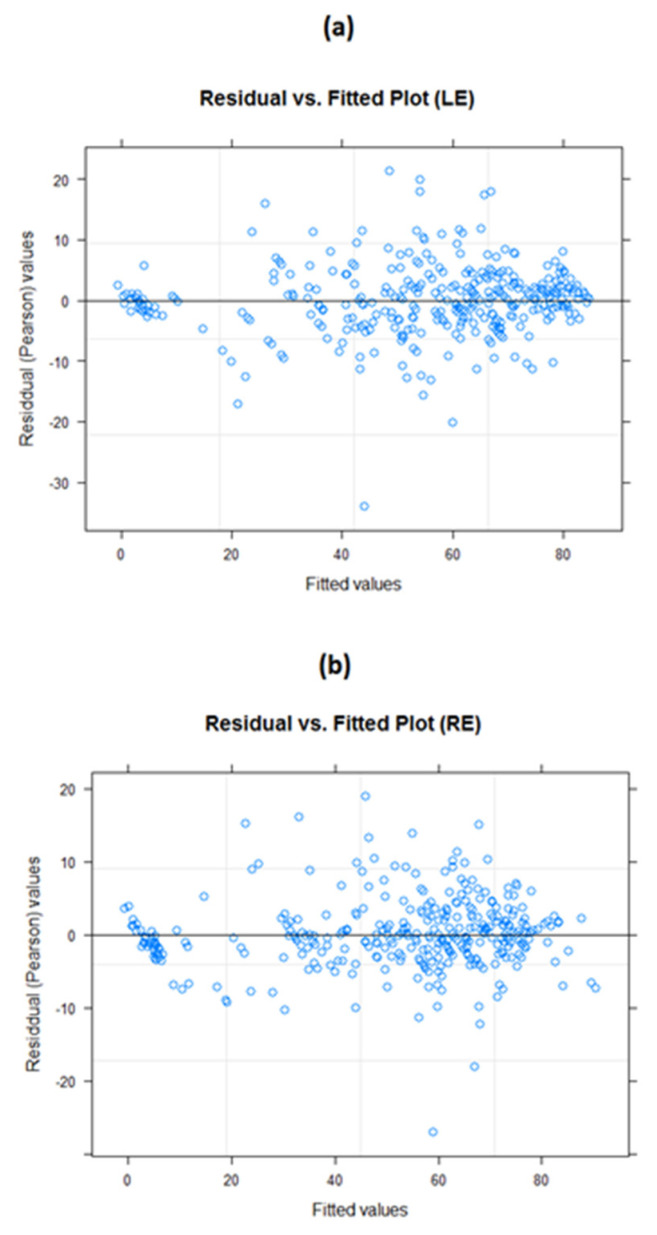
Residual versus fitted value plots. (**a**) LE model and (**b**) RE model. The residual plots appear to be evenly distributed, with no particular patterns emerging; this suggests the models are generally good fits to the data.

**Figure 5:**
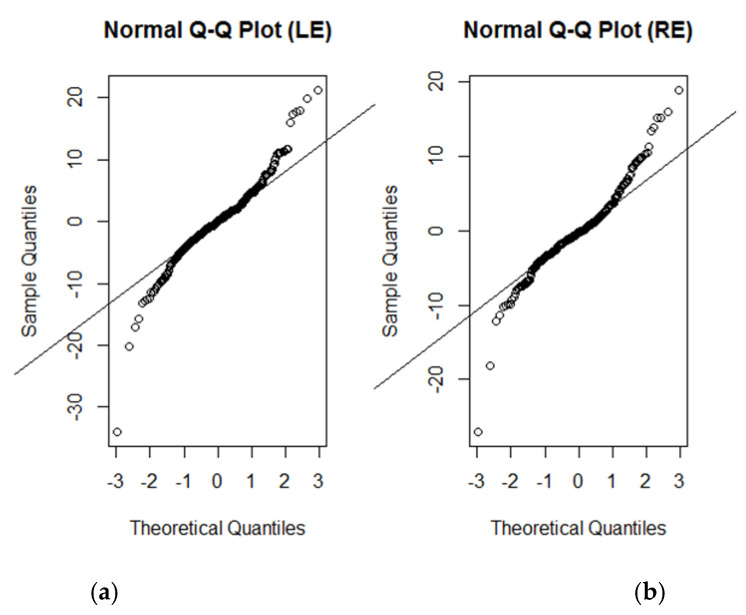
Normal probability plot of residuals. (**a**) LE model and (**b**) RE model. The normal probability plot of residuals appears to be generally and normally distributed, except for some deviation around the tails.

Residual versus fitted plots for both LE and RE models (Figure 4) demonstrated a relatively even distribution. Some data points which were located considerably further out than most other data points could be considered potential outliers. The normal probability plots (Figure 5) for both these models were generally normally distributed, with some deviation noted at the tail ends. While these plots suggested that the models were a good fit for the data, we must consider the possibility of influential data points.

**Figure 6:**
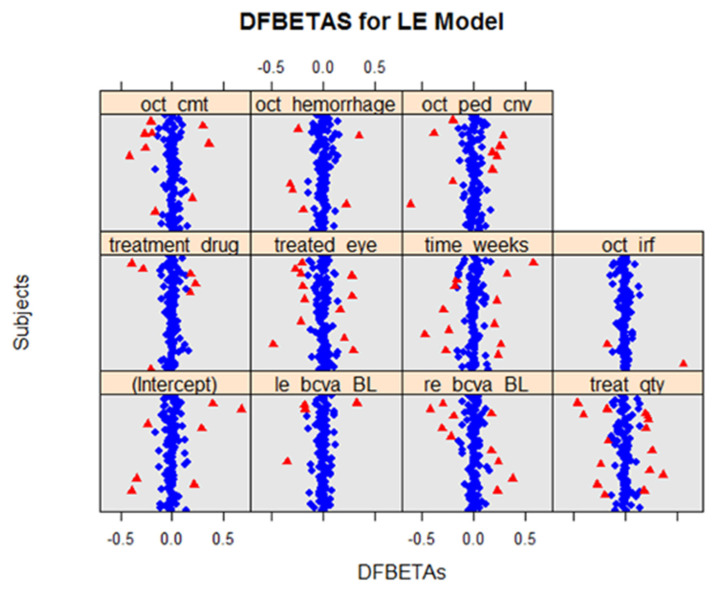
*DFBETAS* for LE models for all variables. Using the cut-off value of $2/\sqrt{n}$, our plot suggests that there are several potential influential points (indicated in red). However, using *sigtest()*,we found that the removal of the *DFBETAS* had no bearing on the model outcomes.

**Figure 7:**
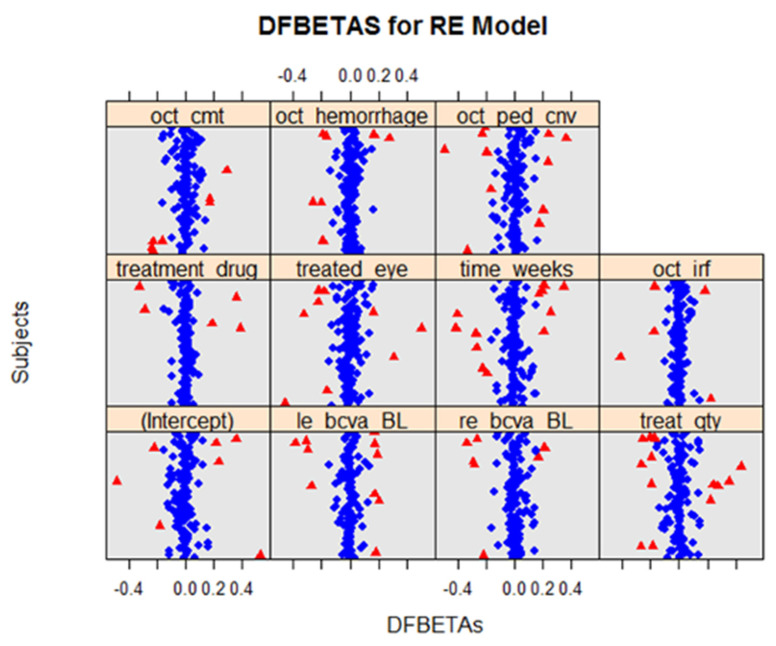
*DFBETAS* for RE models for all variables. Using the cut-off value of $2/\sqrt{n}$, the plots suggested that there are several potential influential points (indicated in red). However, using *sigtest()*, we found that the removal of the *DFBETAS* had no bearing on the model outcomes.

Using *DFBETAS* plots for both the LE (Figure 6) and RE (Figure 7) models, several data points for both models exceeded the cut-off value of $\frac{2}{\sqrt{n}}=0.17$. Using the *sigtest()*, which identified the statistical changes in the model that may be caused by the removal of a potentially influential data point, the removal of the *DFBETAS,* which exceeded the cut-off values, did not cause changes in the outcome for either the LE or RE models.

**Figure 8:**
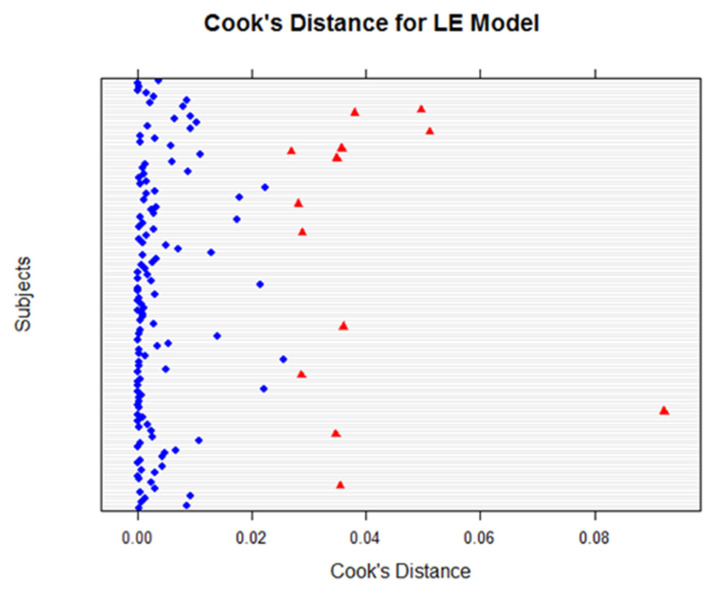
Cook’s distance for LE models for all variables. Using the cut-off value of 4/n, the plot revealed potential influential points (indicated in red). Statistical tests revealed the impacts were not significant.

**Figure 9:**
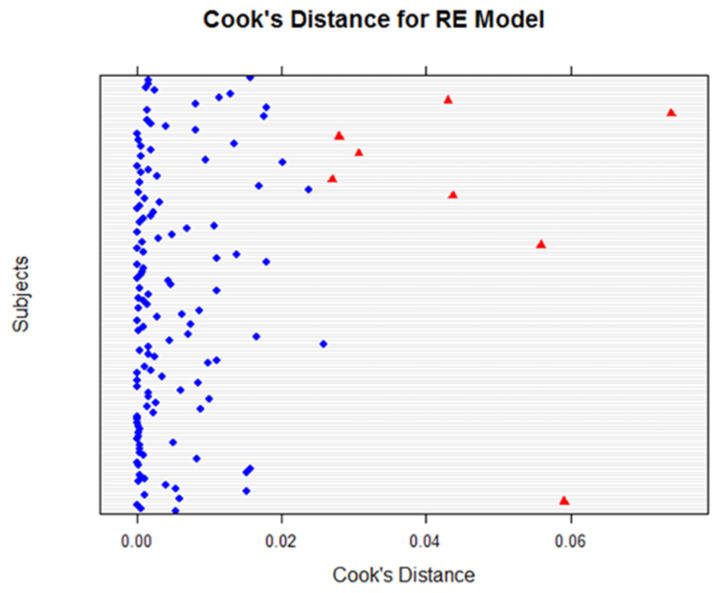
Cook’s distance for RE models for all variables. Using the cut-off value of 4/n, the plot reveals potential influential points (indicated in red), but the tests revealed that there was no significant impact.

Using Cook’s distance plots for both the LE (Figure 8) and RE (Figure 9), several plot points exceeded the cut-off $\frac{4}{n}=0.027$. We reviewed these points by momentarily excluding them using *exclude.influence()* and re-assessing our models; we found that the exclusion of these points did not affect or change our model outcomes.

**Figure 10:**
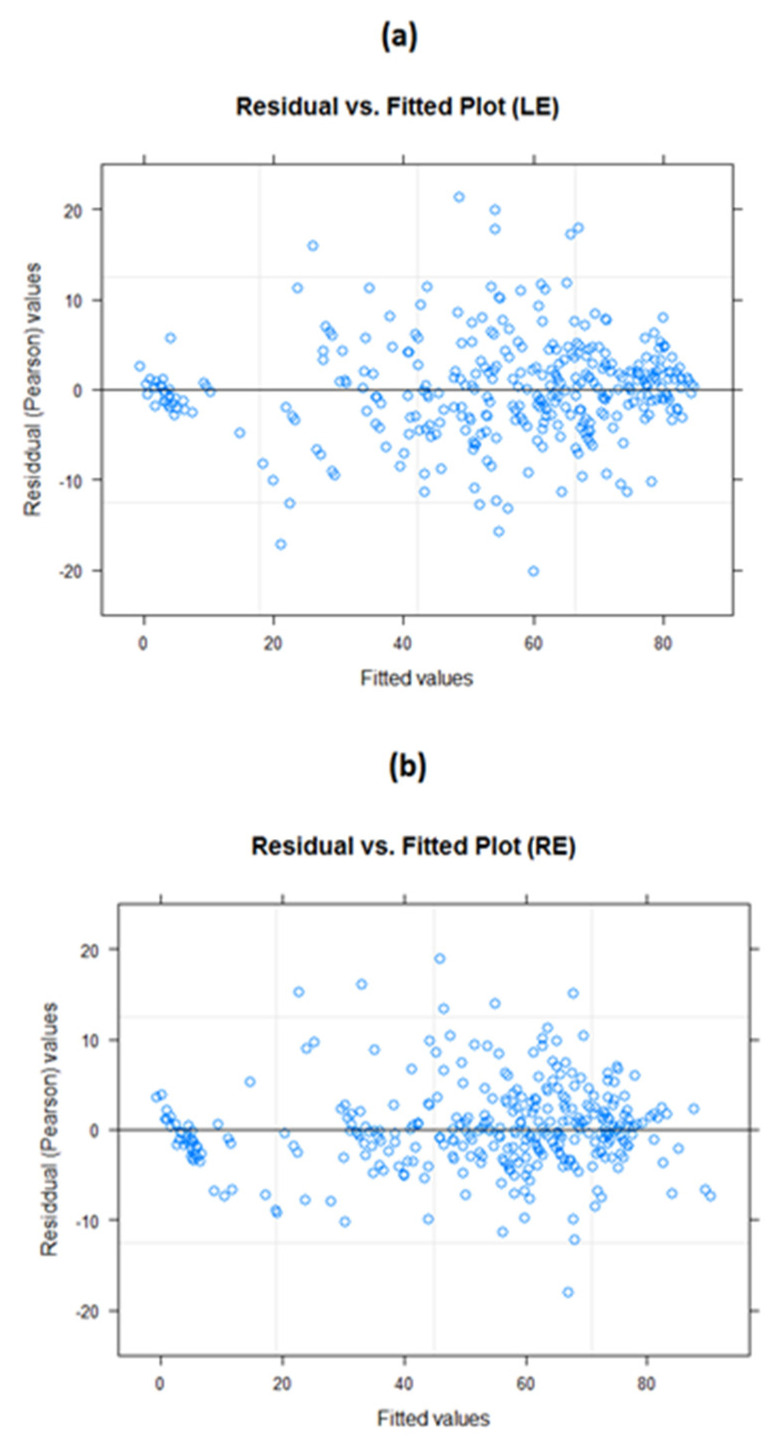
Residual versus fitted value plot. (**a**) LE model and (**b**) RE model without outliers. It is evident that the model assumptions include evenly distributed and randomly spaced plot points.

These results suggest that, while there are several potentially influential data points, no data points appeared to be overly influential on our models. Additionally, we noticed that the original outliers we had noted in the residual versus fitted plots (Figure 4) appeared in our potentially influential analysis. However, similar to all the other potential data points, we noticed that the originally identified outliers had no bearing on the model (or prediction) accuracies. While we opted not to delete outlier points for posterity, we modified the dimensions of the residual versus fitted plots to demonstrate that, sans the outliers, we could clearly see evenly distributed and well-spaced data points of our residual plot (Figure 10), further validating that our model assumptions had been met.

#### 3.2.3. Prediction Accuracy

The forecasting accuracy for the prediction model was evaluated for both the LE and RE models (Table 2). Very low ME results were evident in both LE and RE models. Both sets of MAD results were quite low, with the LE model having a MAD of 1.70–1.87 and the RE model with a MAD value of 1.48–1.55. The RMSE ranged from 3.54 to 3.95 for the LE model and from 3.54 to 3.95 for the RE model.

With respect to the MPE and MAPE, the aim was to identify models which had a MAPE of less than 10%. MAPE for the LE model ranged from 5.56 to 6.39%, and for the RE model, from 7.02 to 7.41. Both models met the MAPE objective. Both LE and RE model MPE results were very low, being −0.02 and −0.03, respectively.

**Figure 11:**
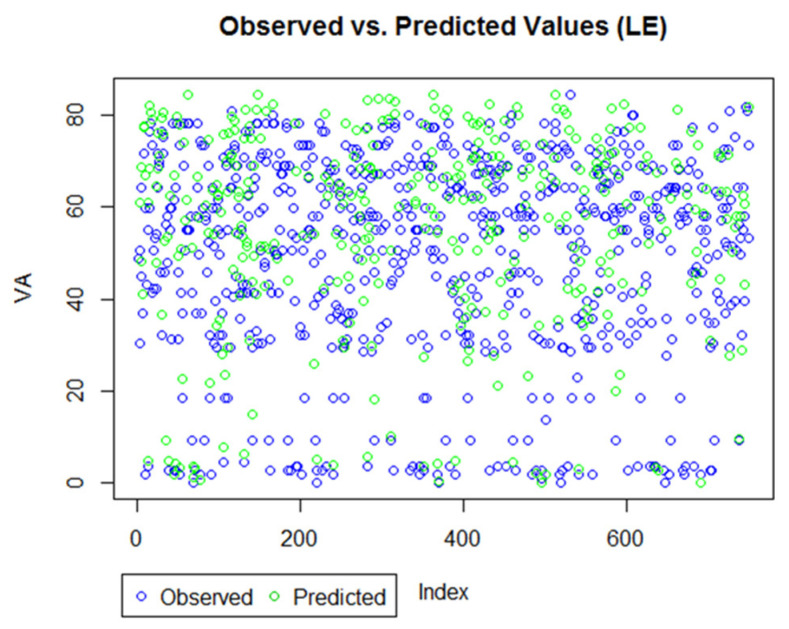
Observed versus predicted value for LE model. The plot suggests most observed and predicted values are overlapping, suggesting a good prediction technique.

**Figure 12:**
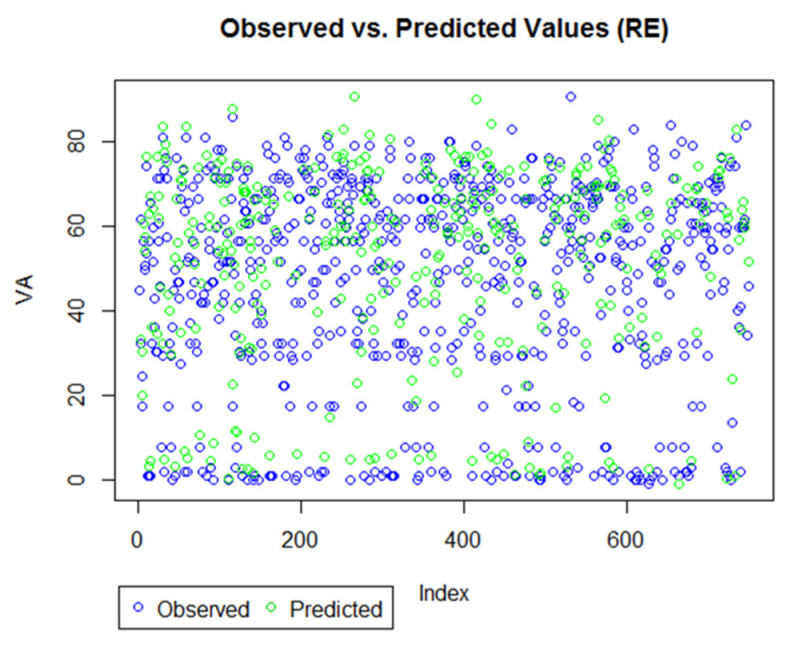
Observed versus predicted value for RE model. The plot shows most observed and predicted values are overlapping, suggesting a good prediction technique.

Finally, for goodness-of-fit, which included both the marginal and conditional $R^{2},$ both models had values close to 1, suggesting that the models were a good fit to the data. Figure 11 and Figure 12 provide a visual demonstration of the proximity between the observed and predicted values. The forecasting errors, along with the visual aids, suggest that the models, in general, have very good prediction accuracy, and the approach is suitable for predicting VA outcomes during anti-VEGF treatment for AMD patients.

### 3.3. Relative Variables of Importance

We computed two sets of RIV analyses: (1) for all nineteen variables that were available (i.e., clinical variables available to ophthalmologists) and (2) for the ten predictor variables found only in our LE and RE models. We ran analyses across the five imputed datasets produced using Amelia. RIVs were weighted for both the LE (Table 3 for the full list of variables; Table 4 for model-only variables) and RE (Table 5 for the full list of variables; Table 6 for model-only variables), with the outcome set as the follow-up VA scores over the course of 24 months.

Generally, results across all the imputed datasets were consistently similar. We did, however, note a single anomaly in the LE outcomes (Table 3): the IRF in the fifth imputed dataset had a $w_{+}$ of 0.77 and $w_{-}$ of 0.23, which contrasted with the previous four imputed dataset outcomes. We repeated our analysis for this measure, and the weight scores remained the same. To account for any uncertainties, we averaged the results across all five imputed sets for each variable.

Once averaged, the weights were identified for each eye, and the variables were then ranked based on their average weighted scores across both eyes (Table 7 for the full list of variables; Table 8 for model-only variables). The top four variables were always classified as “Highly Important” and with average $w_{+}$ scores of at least 0.9 were: the treated eye, the baseline VA of the treated eye, the time (measured in weeks), and the number of injections received throughout the 24 months. No variables were classified as “Important”, which included weight scores of between ≥0.7 and <0.9.

For the full list of variables, four variables were identified as “Moderate” based on a weighted score of between ≥0.5 and <0.7; these were: age, smoking status, the treatment drug, and CMT. It is worth noting that the moderate score for the treatment may purely be due to the use of either ranibizumab or bevacizumab in our studies; both anti-VEGFs were categorised as having similar treatment profiles. Diabetes and the baseline VA of the untreated eye were classified as “Low to Moderate” in importance based on weight scores of between ≥0.4 and < 0.5. Finally, variables with the lowest ranks (i.e., $w_{+}<0.4$) were gender, IRF, SRF, haemorrhage, PED, smokerpacks, hypertension, and ethnicity (both maternal and paternal).

For the model-only variables, those that were identified as “Moderate” included CMT and PED. Those in the “Low to Moderate” categories were the baseline VA of the untreated eye, and IRF. Treatment drug and haemorrhage in this instance was noted as being “Low.”

When comparing the rank of variables between the full list of variables available and those of our model, we noticed for the most part the rank/order of the variables were similar. Minor differences were evident. However, this is unsurprising given that the RIV method ranks variables as relative to the presence of other variables. Overall, though, the rank/order generally appears to remain the same across the board.

## 4. Discussion

Many AMD patients have variable responses to anti-VEGF injections due to medical issues, lifestyle, and demographic factors. A machine learning approach was developed for the prediction of VA outcomes that accounted for these modifying factors and also ranked the predictors in order of importance. The prediction model included age, treated baseline VA, the time of treatment, treatment quantity, the treated eye, baseline of the untreated eye, treatment drug, CMT, IRF, PED, and haemorrhage.

The analytic approach combined a mixed-effects (ME) model and RIV methods, together with the treatment of missing values with the multiple imputation (MI) method and various statistical diagnostic tests to confirm the validity of the model assumptions, such as the normality of residuals.

The variables with the highest rankings included the baseline VA of the treated eye, the time of treatment, treatment quantity, and the treated eye. Given that these variables are important aspects of the anti-VEGF response, their high rankings are unsurprising. The presence of variables, such as age, hypertension, and SRF, had a less significant impact on the accuracy of the model predictions of VA. The analytic approach had a number of strengths and weaknesses, which are described as follows.

### 4.1. Strengths of the Study

Incorporating mixed-effects modelling as part of a machine learning approach is consistent with the analysis of biological and medical data [31], as it provides flexible and powerful statistical tools for controlling stratification, relatedness, and confounding factors [32,33,34]. Features that support statistical confidence in the methodology include the use of the ME and RIV methods to aid in the assessment of predictor importance and the multiple imputation (MI) treatment of missing values. Statistical diagnostics produced very good support for the model with respect to the analysis of residuals and outliers, using methods such as Q–Q plots and Cook’s distance.

There were two noteworthy features of the machine learning approach described in this investigation. First, the use of *time* as an explicit variable in the model is often absent in other machine learning approaches, especially in classification studies comparing training data with test data. This means that no assumptions were necessary on the issue of non-stationarity in the time-series statistics for function approximation, and there was no confounding of the time in either the training or test data, thus reducing error and uncertainty.

The second feature of note is that the weighting and ranking of predictors, as described by the methods in this study, provides information on the relative impact of each predictor on visual acuity and, therefore, adds a degree of *explainability* to the results. In machine learning research, there is currently a strong interest in improving explainability in order to reveal the reasoning used in decision-making and to avoid a black-box analysis by AI algorithms [38]. In the case of explainable AI research, there is a class of approaches commonly referred to as ‘attribution’ methods, which assign to each input feature a score representing its contribution to the response function [85,86]. The machine learning method in this study is an example of such an attribution approach.

### 4.2. Limitations of the Study

The study also has several limitations. With respect to the collection of clinical data. The data were collected retrospectively, and the treatment protocol varied according to a clinician’s choice. The cohort was collected early in the history of anti-VEGF treatment, and as such, individual clinician treatment protocols may have evolved in more recent cases. Similarly, the OCT quality was lower compared to the current advances in spectral domain OCT technology. As such, the ability to judge the presence of SRF and IRF scarring was not as accurate as it could have been if the cohort had been collected more recently. Missing data, particularly relating to retinal characteristics identified by OCT, were most likely due to poor-quality OCT images.

To account for the missing data, we created both multiple and separately imputed and stacked imputed versions of the original dataset, with the latter being created for model validation purposes. The objective of the multiply imputed datasets was to account for uncertainty by generating imputed values that not only mimicked the distribution of the original data but were also slightly different for each imputed dataset to account for any potential uncertainty. Our second limitation was the use of RIV itself. We assessed these variables as relative to each other; their values may have changed if they were tested against other, stronger predictor variables.

We believe that the model weights $w_{i}$ summed over all the models that included a given variable provided a better weight of evidence for the importance of each variable in the context of the set models considered. Using the predictor variables that were considered of interest, the rank of the aforementioned predictors (Table 5 and Table 8) provided a good indication of the relative importance of the variables considered in determining the treatment response. However, with improved imaging technology, new variables, and new data, it is feasible that the relative importance of some variables may need updating—which can be accomplished using the proposed approach.

## 5. Conclusions

This study developed a methodology and prediction model for visual acuity (VA) response following anti-VEGF therapy in nAMD patients. The analysis provided an approach for targeting and prioritising contextual factors that may have an impact on the degree of success in the treatment of wet AMD with anti-VEGF treatments. The evaluation of visual responses included the assessment of clinical, lifestyle, and demographic factors. The approach combined mixed-effects modelling with the relative importance of variables (RIV) modelling, together with statistical learning approaches and data processing with diagnostic tests. The most important predictors were confirmed as the baseline VA, time to treatment, treatment quantity, and the treated eye involved. There were also impacts from OCT features, such as CMT, IRF, PED, and the presence of haemorrhage, together with lifestyle and demographic factors, such as age and ethnicity.

There are several noteworthy features of the study. The incorporation of mixed-effects modelling as part of the machine learning approach is compatible with the analysis of biological and medical data. The approach provided powerful statistical tools for controlling stratification, relatedness, and confounding factors. Statistical confidence in the methodology is highlighted by the use of mixed-effects modelling and RIV methods for the assessment of predictor importance and the multiple imputation (MI) treatment of missing values. Statistical diagnostics underpinned the model performance with respect to the analysis of residuals and outliers, using methods such as Q–Q plots and Cook’s distance.

The study provided support for the use of machine learning in personalised medicine. The machine learning approach investigated had some notable attributes. First, the use of time as an explicit variable avoids issues of non-stationarity and confounding in statistics that may be a problem in classification studies. Second, the approach had a degree of explainability because of its inclusion of attribution analysis.

The flexibility of the approach allowed for extending the model to investigate other potential predictors from personal electronic health records and also updating weights with new training data.

## Figures and Tables

**Table 1:** Summary statistics: patient demographics and clinical characteristics collected at baseline.

**Sex, n (%)**	
Female	85 (56.7)
Male	65 (43.3)
**Age (years)**	
Mean ± SD	78.9 ± 7.3
Range	54–102
**Baseline VA, LE**	
Mean ± SD	53.5 ± 24.0
Range	0–88
**Baseline VA, RE**	
Mean ± SD	48.4 ± 24.3
Range	2–90
**Number of injections at baseline, *n* (%)**	
Ranibizumab	122 (81.3)
Bevacizumab	28 (18.7)
**Smoking Status, *n* (%)**	
No	53 (35.3)
Yes—Past	64 (42.7)
Yes—Present	19 (12.7)
Yes—Virtually Never	8 (5.3)
Missing	6 (4.0)
**Smoker Packs (years)**	
Mean ± SD	39.1 ± 28.7
Range	2–126
**Treated Eye, *n* (%)**	
LE	64 (42.7)
RE	86 (57.3)
**Hypertension, *n* (%)**	
No	48 (32)
Yes	102 (68)
**Diabetes, *n* (%)**	
No	118 (78.7)
Yes	25 (16.7)
Missing	7 (4.6)
**OCT IRF, *n* (%)**	
Absent	24 (16)
Present	85 (56.7)
Missing	41 (27.3)
**OCT SRF, *n* (%)**	
Absent	24 (16)
Present	84 (56)
Missing	42 (28)
**OCT PED, *n* (%)**	

**Table 2:** Metrics for model performance.

	ME	MAD	RMSE	MPE	MAPE (%)	Marginal R^2^	Conditional R^2^
**LE Model**	0.016	1.70	3.54	−0.02	5.56	0.80	0.92
	0.004	1.87	3.94	−0.02	6.37	0.80	0.92
	−0.002	1.87	3.95	−0.02	6.39	0.80	0.92
**RE Model**	−0.002	1.48	3.54	−0.03	7.02	0.75	0.95
	0.016	1.53	3.94	−0.03	7.01	0.75	0.95
	−0.005	1.55	3.95	−0.03	7.41	0.75	0.95

**Table 3:** Relative variables of importance across five imputed datasets for treated LE of all variables.

Variable	Weights	1st Imputed Data	2nd Imputed Data	3rd Imputed Data	4th Imputed Data	5th Imputed Data	Average
**Age**	$w_{+}$	0.33	0.32	0.31	0.34	0.35	0.33
	$w_{-}$	0.67	0.68	0.69	0.66	0.65	0.67
**Baseline VA (LE)**	$w_{+}$	1	1	1	1	1	1
	$w_{-}$	0	0	0	0	0	0
**Baseline VA (RE)**	$w_{+}$	0.42	0.39	0.43	0.42	0.4	0.412
	$w_{-}$	0.58	0.61	0.57	0.58	0.6	0.588
**CMT**	$w_{+}$	0.35	0.27	0.31	0.33	0.31	0.314
	$w_{-}$	0.65	0.73	0.69	0.67	0.69	0.686
**Diabetes**	$w_{+}$	0.71	0.61	0.61	0.73	0.71	0.674
	$w_{-}$	0.29	0.39	0.39	0.27	0.29	0.326
**Ethnicity (maternal)**	$w_{+}$	0.32	0.32	0.34	0.27	0.31	0.312
	$w_{-}$	0.68	0.68	0.66	0.73	0.69	0.688
**Ethnicity (paternal)**	$w_{+}$	0.32	0.31	0.37	0.29	0.29	0.316
	$w_{-}$	0.68	0.69	0.63	0.71	0.71	0.684
**Gender**	$w_{+}$	0.3	0.31	0.38	0.32	0.3	0.322
	$w_{-}$	0.7	0.69	0.62	0.68	0.7	0.678
**Haemorrhage**	$w_{+}$	0.29	0.29	0.27	0.27	0.29	0.282
	$w_{-}$	0.71	0.71	0.73	0.73	0.71	0.718
**Hypertension**	$w_{+}$	0.35	0.32	0.29	0.33	0.33	0.324
	$w_{-}$	0.65	0.68	0.71	0.67	0.67	0.676
**IRF**	$w_{+}$	0.33	0.29	0.34	0.27	0.77	0.4
	$w_{-}$	0.67	0.71	0.66	0.73	0.23	0.6
**PED**	$w_{+}$	0.27	0.5	0.77	0.27	0.38	0.438
	$w_{-}$	0.73	0.5	0.23	0.73	0.62	0.562
**Smokerpacks**	$w_{+}$	0.28	0.3	0.28	0.28	0.3	0.288
	$w_{-}$	0.72	0.7	0.72	0.72	0.7	0.712
**Smoking status**	$w_{+}$	0.49	0.43	0.45	0.39	0.44	0.44
	$w_{-}$	0.51	0.57	0.55	0.61	0.56	0.56
**SRF**	$w_{+}$	0.27	0.34	0.37	0.35	0.31	0.328
	$w_{-}$	0.73	0.66	0.63	0.65	0.69	0.672
**Time (weeks)**	$w_{+}$	1	1	1	1	1	1
	$w_{-}$	0	0	0	0	0	0
**Treated eye**	$w_{+}$	0.99	0.99	0.99	0.99	0.96	0.984
	$w_{-}$	0.01	0.01	0.01	0.01	0.04	0.016
**Treatment drug**	$w_{+}$	0.27	0.33	0.42	0.27	0.28	0.314
	$w_{-}$	0.73	0.67	0.58	0.73	0.72	0.686
**Treatment quantity**	$w_{+}$	0.91	0.85	0.95	0.88	0.96	0.91
	$w_{-}$	0.09	0.15	0.05	0.12	0.04	0.09

CMT: Central macular thickness; IRF: Intra-retinal fluid; PED: Pigment epithelium detachment; SRF: Sub-retinal fluid; VEGF: Vascular endothelial growth factors.

**Table 4:** RIV across five imputed datasets for LE prediction model only.

Variable	Weights	1st Imputed Dataset	2nd Imputed Dataset	3rd Imputed Dataset	4th Imputed Dataset	5th Imputed Dataset	Average
**Treated eye**	$w_{+}$	1	0.99	0.99	0.99	0.99	0.99
	$w_{-}$	0	0.01	0.01	0.01	0.01	0.01
**Baseline VA (LE)**	$w_{+}$	1	1	1	1	1	1
	$w_{-}$	0	0	0	0	0	0
**Time (weeks)**	$w_{+}$	1	1	1	1	1	1
	$w_{-}$	0	0	0	0	0	0
**Treatment quantity**	$w_{+}$	0.91	0.84	0.84	0.84	0.84	0.85
	$w_{-}$	0.09	0.16	0.16	0.16	0.16	0.15
**Treatment Drug**	$w_{+}$	0.3	0.29	0.29	0.29	0.29	0.29
	$w_{-}$	0.7	0.71	0.71	0.71	0.71	0.71
**IRF**	$w_{+}$	0.51	0.54	0.54	0.54	0.54	0.53
	$w_{-}$	0.49	0.46	0.46	0.46	0.46	0.47
**CMT**	$w_{+}$	0.29	0.27	0.27	0.27	0.27	0.27
	$w_{-}$	0.71	0.73	0.73	0.73	0.73	0.73
**Haemorrhage**	$w_{+}$	0.32	0.46	0.46	0.46	0.46	0.43
	$w_{-}$	0.68	0.54	0.54	0.54	0.54	0.57
**Baseline VA (RE)**	$w_{+}$	0.41	0.4	0.4	0.4	0.41	0.40
	$w_{-}$	0.59	0.6	0.6	0.6	0.59	0.60
**PED**	$w_{+}$	0.93	0.65	0.65	0.65	0.65	0.71
	$w_{-}$	0.07	0.35	0.35	0.35	0.35	0.29

CMT: Central macular thickness; IRF: Intra-retinal fluid; PED: Pigment epithelium detachment; SRF: Sub-retinal fluid; VEGF: Vascular endothelial growth factors.

**Table 5:** Relative variables of importance across five imputed datasets for treated RE for all variables.

Variable	Weight	1st Imputed Dataset	2nd Imputed Dataset	3rd Imputed Dataset	4th Imputed Dataset	5th Imputed Dataset	Average
**Age**	$w_{+}$	0.87	0.86	0.87	0.78	0.7	0.816
	$w_{-}$	0.13	0.14	0.13	0.22	0.3	0.184
**Baseline VA (RE)**	$w_{+}$	1	1	1	1	1	1
	$w_{-}$	0	0	0	0	0	0
**Baseline VA (LE)**	$w_{+}$	0.3	0.28	0.28	0.28	0.31	0.29
	$w_{-}$	0.7	0.72	0.72	0.72	0.69	0.71
**CMT**	$w_{+}$	1	1	1	1	0.99	0.998
	$w_{-}$	0	0	0	0	0.01	0.002
**Diabetes**	$w_{+}$	0.26	0.26	0.37	0.28	0.26	0.286
	$w_{-}$	0.74	0.74	0.63	0.72	0.74	0.714
**Ethnicity (maternal)**	$w_{+}$	0.35	0.38	0.39	0.37	0.34	0.366
	$w_{-}$	0.65	0.62	0.61	0.63	0.66	0.634
**Ethnicity (paternal)**	$w_{+}$	0.4	0.45	0.44	0.42	0.42	0.426
	$w_{-}$	0.6	0.55	0.56	0.58	0.58	0.574
**Gender**	$w_{+}$	0.3	0.27	0.3	0.29	0.29	0.29
	$w_{-}$	0.7	0.73	0.7	0.71	0.71	0.71
**Haemorrhage**	$w_{+}$	0.34	0.27	0.34	0.27	0.26	0.296
	$w_{-}$	0.66	0.73	0.66	0.73	0.74	0.704
**Hypertension**	$w_{+}$	0.28	0.29	0.27	0.28	0.27	0.278
	$w_{-}$	0.72	0.71	0.73	0.72	0.73	0.722
**IRF**	$w_{+}$	0.28	0.26	0.27	0.27	0.34	0.284
	$w_{-}$	0.72	0.74	0.73	0.73	0.66	0.716
**PED**	$w_{+}$	0.26	0.3	0.27	0.28	0.48	0.318
	$w_{-}$	0.74	0.7	0.73	0.72	0.52	0.682
**Smokerpacks**	$w_{+}$	0.36	0.45	0.38	0.47	0.53	0.438
	$w_{-}$	0.64	0.55	0.62	0.53	0.47	0.562
**Smoking status**	$w_{+}$	0.74	0.73	0.82	0.49	0.31	0.618
	$w_{-}$	0.26	0.27	0.18	0.51	0.69	0.382
**SRF**	$w_{+}$	0.27	0.26	0.27	0.27	0.36	0.286
	$w_{-}$	0.73	0.74	0.73	0.73	0.64	0.714
**Time (weeks)**	$w_{+}$	1	1	1	1	1	1
	$w_{-}$	0	0	0	0	0	0
**Treated eye**	$w_{+}$	1	1	1	1	1	1
	$w_{-}$	0	0	0	0	0	0
**Treatment drug**	$w_{+}$	0.95	1	0.43	0.58	0.98	0.788
	$w_{-}$	0.05	0	0.57	0.42	0.02	0.212
**Treatment quantity**	$w_{+}$	1	1	1	1	1	1
	$w_{-}$	0	0	0	0	0	0

CMT: Central macular thickness; IRF: Intra-retinal fluid; PED: Pigment epithelium detachment; SRF: Sub-retinal fluid; VEGF: Vascular endothelial growth factors.

**Table 6:** RIV across five imputed datasets for RE prediction model only.

Variable	Weights	1st Imputed Dataset	2nd Imputed Dataset	3rd Imputed Dataset	4th Imputed Dataset	5th Imputed Dataset	Average
**Treated eye**	$w_{+}$	1	1	1	1	1	1
	$w_{-}$	0	0	0	0	0	0
**Baseline VA (LE)**	$w_{+}$	0.3	0.27	0.27	0.27	0.27	0.28
	$w_{-}$	0.7	0.73	0.73	0.73	0.73	0.72
**Time (weeks)**	$w_{+}$	1	1	1	1	1	1
	$w_{-}$	0	0	0	0	0	0
**Treatment quantity**	$w_{+}$	1	1	1	1	1	1
	$w_{-}$	0	0	0	0	0	0
**Treatment Drug**	$w_{+}$	0.49	0.44	0.44	0.44	0.44	0.45
	$w_{-}$	0.51	0.56	0.56	0.56	0.56	0.55
**IRF**	$w_{+}$	0.29	0.27	0.27	0.27	0.27	0.27
	$w_{-}$	0.71	0.73	0.73	0.73	0.73	0.73
**CMT**	$w_{+}$	1	1	1	1	1	1
	$w_{-}$	0	0	0	0	0	0
**Haemorrhage**	$w_{+}$	0.29	0.33	0.33	0.33	0.33	0.32
	$w_{-}$	0.71	0.67	0.67	0.67	0.67	0.68
**Baseline VA (RE)**	$w_{+}$	1	1	1	1	1	1
	$w_{-}$	0	0	0	0	0	0
**PED**	$w_{+}$	0.28	0.32	0.32	0.32	0.32	0.31
	$w_{-}$	0.72	0.68	0.68	0.68	0.68	0.69

CMT: Central macular thickness; IRF: Intra-retinal fluid; PED: Pigment epithelium detachment; SRF: Sub-retinal fluid; VEGF: Vascular endothelial growth factors.

**Table 7:** Classification of all 19 variables into groups.

Variables	Level of Importance
Baseline VA of treated eye	Highly Important
Treated eye	Highly Important
Time (weeks)	Highly Important
Number of injections	Highly Important
Age	Moderate
Smoking Status	Moderate
Treatment drug	Moderate
CMT	Moderate
Baseline VA of untreated eye	Low to Moderate
Diabetes	Low to Moderate
Gender	Low
IRF	Low
SRF	Low
Haemorrhage	Low
PED	Low
Smokerpacks	Low
Hypertension	Low
Ethnicity (maternal)	Low
Ethnicity (paternal)	Low

Level of importance was based on the following criteria: Highly important: $w_{+}\geq 0.9$; Important: $w_{+}\geq 0.7$ and <0.9; Moderate $w_{+}\geq 0.5$ and <0.7; Low to Moderate: $w_{+}\geq 0.4$ and <0.5; Low: $w_{+}<0.4$.

**Table 8:** Rank of all nine variables found in LE and RE models.

Variables	Level of Importance
Baseline VA of treated eye	Highly Important
Treated eye	Highly Important
Time (weeks)	Highly Important
Number of injections	Highly Important
CMT	Moderate
PED	Moderate
IRF	Low to Moderate
Baseline VA of untreated eye	Low to Moderate
Haemorrhage	Low
Treatment drug	Low

Level of importance was based on the following criteria: Highly important: $w_{+}\geq 0.9$; Important: $w_{+}\geq 0.7$ and <0.9; Moderate $w_{+}\geq 0.5$ and <0.7; Low to Moderate: $w_{+}\geq 0.4$ and <0.5; Low: $w_{+}<0.4$.

## Data Availability

Not applicable.

## Supplementary Materials

The following supporting information can be downloaded at: https://www.mdpi.com/article/10.3390/life12111926/s1, Table S1: Description of potential predictor variables included in the dataset; Table S2: Potential predictor variables for LE and RE modelling based on multiple imputed datasets; Table S3: Potential predictor variables for LE and RE modelling based on stacked imputed dataset.
