# Supplementary material for: Application of Machine Learning to Ranking Predictors of Anti-VEGF Response
Source: Life (Basel). 2022 Nov 18;12(11):1926. doi: 10.3390/life12111926 (PMC9699567; doi:10.3390/life12111926)
Supplement: Supplementary file 1 [file life-12-01926-s001.zip › life-2002879-supplementary.pdf]

Supplementary Materials

1. Acronyms

AIC: Akaike Information Criteria

AMD: Age-related macular degeneration

Anti-VEGF: Anti-Vascular Endothelial Growth Factors

BIC: Bayesian Information Criteria

CMT: Central macular thickness

CNV: Choroidal neovascularization

EM: Expectation-maximization

ETDRS: Early Treatment Diabetic Retinopathy Study

GA: Geographic Atrophy

IRF: Intra-retinal fluid

LE: Left eye

MAD: Mean Absolute Deviation

MAPE: Mean Absolute Percentage Error

MCMC: Markov Chain Monte Carlo

ME: Mean error

MI: Multiple imputation

MPE: Mean Percentage Error

nAMD: Neovascular AMD.

OCT: Optical coherence tomography

PED: Pigment epithelium detachment

RE: Right eye

RIV: Relative importance of variables

RMSE: Root Mean Square Error

RVEEH: Royal Victorian Ear and Eye Hospital

SHRM: Subretinal hyperreflective material

SRF: Sub-retinal fluid

SD: Standard deviation

VA: Visual Acuity

2. Data Management and Processing

Statistical analysis was carried out using software coded in R version 3.2.2. Treatment of missing values, for MI and stacked MI methods was carried out using the R package *Amelia*. If not stated otherwise, the `lme4()` package due to Bates & Maechler and maintained by Ben Bolker was used in the creation and analysis of the linear mixed-effects models. Variable definitions, types and routines used are referenced in the following tables.

Table S1. Description of potential predictor variables included in the dataset

Variable	Type	Input Name
VA scores at baseline	Continuous	Baseline VA LE: `bcva_le`<br>Baseline VA RE: `bcva_re`
Time of appointment (in weeks)		`time_weeks`
Anti-VEGF treatment protocol	Categorical	`treatment_drug`
Treatment number	Continuous	`treat_qty`
Treated eye	Categorical	`treated_eye`
OCT presence of IRF and SRF	Categorical	`oct_irf; oct_srf`
CMT	Continuous	`oct_cmt`
PED	Binary	`oct_ped_cnv`
Haemorrhage	Binary	`oct_hemorrhage`
Hypertension	Binary	`hypertension`
Diabetes	Binary	`Diabetes`
Smoking status	Categorical	`smoking_status`
Smoker packs	Continuous	`smokerpacks`
Age	Continuous	`Age`
Gender	Binary	`Gender`
Maternal and paternal ethnicity	Categorical	`ethnicity_mom`<br>`ethnicity_dad`

CMT: Central macular thickness; OCT: Optical coherence tomography; IRF: Intra-retinal fluid; PED: Pigment epithelium detachment; SRF: Sub-retinal fluid; VEGF: Vascular Endothelial Growth Factors.

Table S2. Potential predictor variables for LE and RE modelling based on multiple imputed datasets.

Imputed Dataset	Potential Predictor Variables using five imputed datasets
**LE**	
1	le_bcva_BL, diabetes, smoking_status, time_weeks, treat_qty, treated_eye
2	le_bcva_BL, diabetes, oct_ped_cnv, time_weeks, treat_qty, treated_eye
3	le_bcva_BL, diabetes, oct_ped_cnv, time_weeks, treat_qty, treated_eye
4	le_bcva_BL, diabetes, time_weeks, treat_qty, treated_eye
5	le_bcva_BL, diabetes, oct_irf, time_weeks, treat_qty, treated_eye
**RE**	
1	re_bcva_BL, age, oct_cmt, smoking_status, time_weeks, treat_qty, treated_eye, treatment_drug
2	re_bcva_BL, age, oct_cmt, smoking_status, ethnicity_dad, time_weeks, treat_qty, treated_eye, treatment_drug
3	re_bcva_BL, age, oct_cmt, smoking_status, ethnicity_dad, time_weeks, treat_qty, treated_eye
4	re_bcva_BL, age, oct_cmt, smokerpacks, time_weeks, treat_qty, treated_eye, treatment_drug
5	re_bcva_BL, age, oct_cmt, smokerpacks, time_weeks, treat_qty, treated_eye, treatment_drug

Table S3. Potential predictor variables for LE and RE modelling based on stacked imputed dataset.

	Potential predictor variables identified using the stacked imputation method
**LE**	le_bcva_BL, oct_cmt, oct_irf, oct_ped_cnv, re_bcva_BL, time_weeks, treat_qty, treated_eye, treatment_drug
**RE**	re_bcva_BL, hypertension, age, le_bcva_BL, oct_cmt, oct_hemorrhage, oct_irf, oct_srf, smokerpacks, time_weeks, treat_qty, treatment_drug
